# Adult Goat Retinal Neuronal Culture: Applications in Modeling Hyperglycemia

**DOI:** 10.3389/fnins.2019.00983

**Published:** 2019-09-16

**Authors:** Sapana Sharma, Harshini Chakravarthy, Gowthaman Suresh, Vasudharani Devanathan

**Affiliations:** Department of Biology, Indian Institute of Science Education and Research (IISER), Tirupati, India

**Keywords:** neurodegeneration, hyperglycemia, retinal neurons, CCAAT-enhancer-binding protein, adult neurons, cell adhesion molecules, neurite extension

## Abstract

Culture of adult neurons of the central nervous system (CNS) can provide a unique model system to explore neurodegenerative diseases. The CNS includes neurons and glia of the brain, spinal cord and retina. Neurons in the retina have the advantage of being the most accessible cells of the CNS, and can serve as a reliable mirror to the brain. Typically, primary cultures utilize fetal rodent neurons, but very rarely adult neurons from larger mammals. Here, we cultured primary retinal neurons isolated from adult goat up to 10 days, and established an *in vitro* model of hyperglycemia for performing morphological and molecular characterization studies. Immunofluorescence staining revealed that approximately 30–40% of cultured cells expressed neuronal markers. Next, we examined the relative expression of cell adhesion molecules (CAMs) in adult goat brain and retina. We also studied the effect of different glucose concentrations and media composition on the growth and expression of CAMs in cultured retinal neurons. Hyperglycemia significantly enhances neurite outgrowth in adult retinal neurons in culture. Expression of CAMs such as Caspr1, Contactin1 and Prion is downregulated in the presence of high glucose. Hyperglycemia downregulates the expression of the transcription factor CCAAT/enhancer binding protein (C/EBP α), predicted to bind CAM gene promoters. Collectively, our study demonstrates that metabolic environment markedly affects transcriptional regulation of CAMs in adult retinal neurons in culture. The effect of hyperglycemia on CAM interactions, as well as related changes in intracellular signaling pathways in adult retinal neurons warrants further investigation.

## Introduction

*In vitro* study of adult neurons is a fundamental and indispensable tool for understanding the precise contribution of neuronal genes and proteins toward the pathophysiology of neurodegenerative diseases. Analysis of neurons cultured in isolation over time facilitates perturbation of neuron-specific signaling pathways by exposing them to chemical agents, and manipulation of neuronal genes using knock-down or overexpression studies. Traditionally, neurons are studied by *in vitro* culturing of cells obtained not from adult, but from embryonic tissue or young pups within 1–10 days of birth (Tabata et al., 2000; Liu et al., 2013; Gao et al., 2016), since adult tissue consists of mature neurons which do not undergo cell division. The culture of early postnatal neurons from embryonic or immature tissue has enabled crucial advances in our understanding of molecular pathways involved in development or differentiation (Watanabe and Raff, 1990; Waid and McLoon, 1998; Reese, 2011). However, these cultures are of limited value in studying neurodegenerative disease which primarily affects mature and aged neuronal tissue. Study of hyperglycemia-associated neuronal damage in adult tissues isolated from higher mammals may provide clinically-relevant data applicable to adult-onset diabetes which currently affects nearly half a billion people worldwide. Although fully post-mitotic, terminally differentiated adult neurons retain the ability regenerate their neurites when maintained in culture and hence may be more useful as an *in vitro* model system for investigating neuroprotection, neurite regeneration and pathogenic mechanisms of neurodegenerative disease (Brewer et al., 2005; Ghosh et al., 2012; Salvadores et al., 2017).

Similar to the brain, retinal neurons and Müller glia are derived from the neuroepithelium in two temporal phases during embryonic development (Centanin and Wittbrodt, 2014). In recent years, several studies have demonstrated significant correlations between retinal pathology and neurodegeneration in the brain (Ciudin et al., 2017; Ramirez et al., 2017; Mutlu et al., 2018; Sundstrom et al., 2018). Proteomic analysis of post-mortem diabetic human retinas shows activation of the same pathogenic mediators which are involved in neurodegenerative brain diseases (Sundstrom et al., 2018). Retinal microperimetry demonstrates that retinal sensitivity in diabetic patients correlates significantly with brain neurodegeneration (Ciudin et al., 2017). β-amyloid plaques and phosphorylated tau have recently been detected in retinas of Alzheimer’s disease (AD) patients (den Haan et al., 2018), while α-synuclein aggregates have been detected in retinas of Parkinson’s disease patients (Veys et al., 2019). An ongoing clinical trial (NCT02360527) is currently examining the feasibility of using diabetic retinal neurodegeneration as a biomarker for AD. Such correlations are not surprising, since the neural retina is a brain-derived tissue and shares striking molecular parallels with the brain and spinal cord (Byerly and Blackshaw, 2009). Developmentally and anatomically the retina is an extension of the CNS, and consists of five distinct types of neurons forming a complex neural circuitry that transmits visual signals to the brain. Several features of neurodegeneration reported in the brain have been detected in retinal diseases. Amyloid-beta deposition is found in drusen, the hallmark of age-related macular degeneration, and in glaucoma (Dentchev et al., 2003; Yan et al., 2017). Neuroinflammatory markers are associated with onset of AD as well as diabetic retinopathy (Heppner et al., 2015; Chakravarthy and Devanathan, 2018).

Most experiments in neurobiology are conducted using neurons isolated from rodents; however, mouse models often fall short of recapitulating human pathophysiology. In this study we utilized goat retina as our model system; we postulate that *in vitro* studies in goat could facilitate identification of novel pathways in higher mammals which may not be revealed by rodent-derived cells. Here, we examined the relative expression of neuron-specific cell adhesion molecules (CAMs) in adult goat brain and retina. Further, we cultured primary neurons from the retina isolated from adult goat. We also studied the effect of different glucose concentrations and media compositions on neurite outgrowth and expression of CAMs in cultured adult neurons. Our studies reveal that hyperglycemia significantly enhances neurite extension in cultured retinal neurons, while downregulating expression of specific neuronal CAMs. We further propose that the expression of these CAMs is transcriptionally regulated by CCAAT/enhancer binding proteins (CEBP) α and β which have predicted binding sites upstream of goat CAM gene promoters.

## Materials and Methods

### Isolation of Retina and Brain From Adult Goat and Mouse

Eyes were isolated from adult goat (*Capra hircus*), 2–3 months of age from the local abattoir in Tirupati, India. As per institutional guidelines, we obtained written informed consent from the abattoir owner for participation of animals in this study. After euthanasia, brain and eyeballs were carefully removed and transported to the lab in sterile HBSS (Gibco) on ice. In the biosafety cabinet, the eyeball was immersed in 70% ethanol for 2 min. The eyeball was punctured posterior to the limbus, cut around the circumference of the limbus, and cornea, lens, and sclera were removed. The vitreous was extracted, and retina was detached gently from eye cup, by gently dissociating from the retinal pigmented epithelium. A cut was made at the center of the retina to detach it from the optic nerve head. The dissection was done under sterile conditions in HBSS solution (Supplementary Figure S1). The brain was also dissected under sterile conditions to isolate cerebrum, cerebellum and brain stem. 8-week old male BALB/c mice were euthanized, enucleated, and retina isolated as described above. All procedures involving mice were approved by the Institutional Animal Ethics Committee (IAEC number: IAEC/56/SRU/624/2018).

### Establishment of an Adult Retinal Neuron Model of Hyperglycemia

In this study, retinal cells were cultured in normoglycemia (5 mM glucose) or hyperglycemia (25 mM glucose). A previous study has shown that changes in glucose concentrations during culture preparation significantly affects neuronal viability (Kleman et al., 2008). Hence, retinal tissue was processed, washed, triturated and counted in 5 mM or 25 mM glucose-containing buffer/media, depending on the final glucose concentration used. DMEM (Dulbecco’s Modified Eagle Medium) is a widely used basal medium for maintaining mammalian cell types in culture such as fibroblasts, glia, neurons, and cell lines. DMEM is typically supplemented with 10% fetal bovine serum (FBS). Neurobasal-A medium is a basal medium specifically designed for maintenance of adult neurons without the need for an astrocyte feeder layer, when used with serum-free B-27 supplement (Brewer et al., 1993). A major difference between the two media is their osmolarity: DMEM is 335mOsm, while Neurobasal-A is 260mOsm. We compared the growth of adult goat neurons in both DMEM and Neurobasal-A media. We used five goats to establish the consistency of the culture system. From one adult goat, retina isolated from both eyes were used for primary culture.

The isolated retina was immersed in 5 mM or 25 mM glucose-containing HBSS, cut into small pieces (1–2 mm size), and digested with 0.05% trypsin (Gibco) for 15 min, at 37°C. The reaction was terminated by adding DMEM complete medium: 5 mM or 25 mM glucose DMEM (Gibco) respectively, containing 10% FBS (Gibco), 0.06 g/L L-glutamine, and 1% Penicillin/Streptomycin (Sigma). The resultant suspension was filtered through a 40 μm nylon mesh, centrifuged at 200 g for 5 min, and resuspended in DMEM complete medium (Supplementary Figure S2). Immunopanning was done to remove fibroblast and glial cells: Culture dishes were coated with anti-vimentin antibody (1:50) prepared in 50 mM Tris–HCl. Anti-vimentin-coated dishes were incubated overnight at 4°C. Immediately before use, dishes were rinsed with PBS three times. Retinal cell suspension was incubated in panning dishes for 60 min, and non-adherent cells were collected. Cells were quantified, and viability assessed after staining with Trypan blue, and viewed under an inverted light microscope (Thermo Fisher Scientific). 1.3 × 10^6^cells/well were added to poly-L-Lysine-coated 6-well plates containing 12 × 12 mm coverslips. After 24 h, media was changed to 5 mM or 25 mM glucose DMEM complete medium, or 25 mM glucose Neurobasal-A medium containing 2% B-27 supplement, 1% Penicillin/Streptomycin, 10% FBS and 0.06 g/L L-glutamine. Half medium was renewed every 1–2 days. Cells were observed daily to evaluate morphology, length of neurites, and adherence as described previously (Perry et al., 1997). Cell counts per field, for 6–10 random fields under 10× magnification were recorded every 2 days up to 10 days.

### MTT Assay for Cytotoxicity Evaluation

Cells were seeded into a 24-well plate at a density of 0.05 million cells/well. Cells were grown in 5 mM glucose and 25 mM glucose-containing DMEM medium, and in Neurobasal-A medium (25 mM glucose). Media was changed every alternate day. After 6 days, 50 μl of MTT solution (0.5 mg/ml) was added and plate incubated at 37°C. After 4 h, cells were treated with 500 μl of isopropanol for 20 min at RT. Absorbance at 570 nm was recorded using a microplate reader (Biotek). The experiment was performed in triplicate.

### Antibodies Used

Contactin-1(1:100), Caspr-2(1:200), PrP(1:200) from Sigma, NCAM-1(1:50), Synaptophysin(1:250), GFAP(1:100), Caspr-1(1:200) from Novus, NeuN(1:100) from Abcam.

### Immunocytochemistry

Immunofluorescence staining was performed as described previously (Sytnyk et al., 2002). Cells cultured for 10 days on coverslips were washed in PBS and fixed in 4% formaldehyde for 15 min at 4°C. Cells were washed three times with PBS, blocked in 1% BSA in PBS, and incubated with primary antibodies for 60 min at RT. Corresponding fluorescent labeled secondary antibodies (Alexa Flour 488 and 594, Invitrogen) staining was done for 60 min at RT. Stained cells were mounted using Fluoromount mounting medium containing nuclear stain DAPI (Sigma). Images were acquired using a fluorescence microscope (Olympus).

### Identification of Neurons and Glial Cells

Cells cultured were stained for neuronal (NeuN, Synaptophysin) and glial (GFAP) markers. 6–10 random fields were examined under 10× magnification. Number of neurons and glia were calculated using the formula: percentage of neurons (%) = number of neurons/total number of cells in the field × 100.

### Quantitative Real-Time PCR

Tissue homogenates of retinas and brain were prepared at 4°C using a Potter Elvehjem homogenizer (Biolab Instruments) in lysis buffer (50 mM HEPES, 1 mM EGTA, 1.5 mM MgCl_2_, 150 mM NaCl, 10% glycerol, 1% Triton ×-100) at pH 7.4, containing protease inhibitor (Roche). After 30 min of mild shaking at 4°C, homogenate was centrifuged at 15000 g for 15 min at 4°C. Supernatant containing protein was flash-frozen and stored in −80°C.

RNA was extracted from cell lysates and tissue homogenates using TRIzol (Invitrogen). RNA was quantified using Nanodrop2000 spectrophotometer (Thermo Fisher Scientific) and reverse-transcribed using iScript cDNA synthesis kit (Bio-Rad). Goat and mouse primers were used for checking expression of specific genes by performing quantitative RT-PCR using iTaq Universal SYBR Green supermix (Bio-Rad). Results were normalized to GAPDH or β-actin. Primers used are given in Supplementary Table S1.

### Neurite Outgrowth Assay

Cultured cells maintained for 10 days were fixed with 4% formaldehyde and immunostained with neuronal marker, NeuN (green). To quantify neurite outgrowth, neurons were visualized under 10× magnification and total length of all neurites were determined by neurite tracing using ImageJ, as described previously (Ng et al., 2003). Neurite length measurements from at least 100 cells per dish were recorded from randomly chosen fields. Each experiment was repeated three times.

### Prediction of C/EBP Binding Sites in CAM Gene Promoters

C/EBP binds to an extensive range of DNA sequences. Several C/EBP-α binding sites were predicted within 600 bp upstream of goat CAM promoters (Caspr1, Caspr2, Prion and Contactin1), using Alibaba2 program, Version 2.1, Germany. Alibaba2 software predicts binding sites of transcription factors in a DNA sequence, available on http://gene-regulation.com/pub/programs/alibaba2/index.html (Grabe, 2002). By convention, numbering of nucleotides on the gene sequence begins with “1” at **A** of ATG start codon, while nucleotides upstream (5’UTR) of ATG-translation initiation codon are marked with a “−” (minus) sign, and are numbered −1, −2, −3, etc. going further upstream from ATG.

### Statistical Analysis

Data are presented as mean ± SEM. Results were analyzed for statistical significance by Student’s *t*-test or one-way ANOVA followed by Tukey’s *post hoc* test (Prism 8, GraphPad Prism).

## Results

### Growth and Morphology of Retinal Neurons in Primary Culture

**FIGURE 1:**
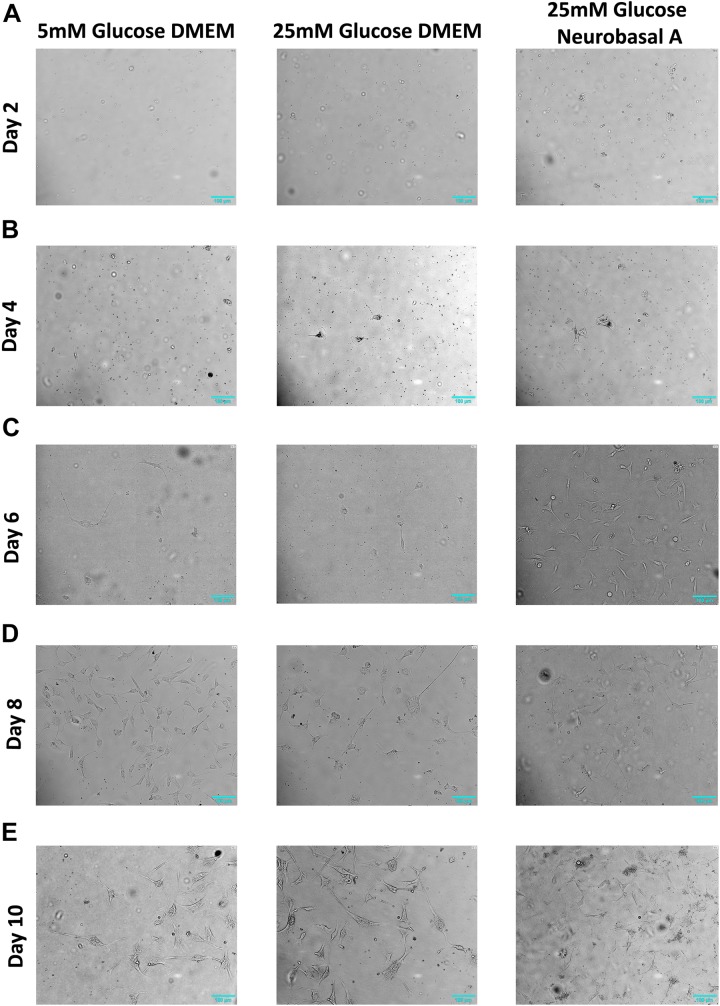
Morphology of retinal neurons cultured in 5 mM glucose DMEM, 25 mM glucose DMEM, or 25 mM glucose Neurobasal A medium. **(A)** On Day 2, adherent cells are few in number in all three media conditions. **(B)** On Day 4, more cells start to attach to the cell culture dish in 25 mM glucose- containing DMEM and Neurobasal A medium, whereas fewer cells are adherent in 5 mM glucose containing DMEM. **(C)** On Day 6, more cells start to adhere to the culture dish, especially in high glucose-containing Neurobasal A medium. **(D)** Day 8 adherent cells continue to grow in culture. **(E)** On Day 10 post primary culture initiation, axon-like projections were prominent in length, gradually forming complex networks. *N* = 5.

**FIGURE 2:**
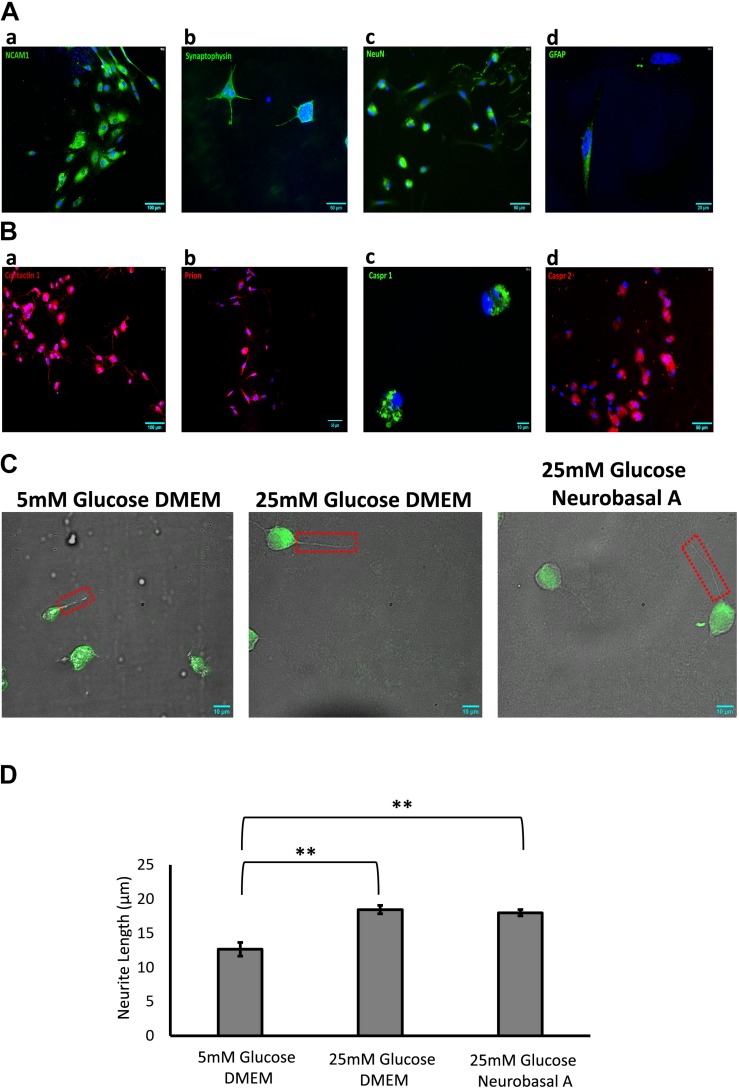
**(A)** Identification of adult retinal neurons and glia in culture by immunofluorescence staining. **(a)** NCAM1 (green), expressed by all retinal neurons and glia; **(b)** Synaptophysin (green), expressed by neurons in the plexiform layers of the retina; **(c)** NeuN (green), expressed by neuronal nuclei; **(d)** GFAP (green), expressed by retinal glia. **(B)** Expression of cell adhesion molecules (CAMs) in adult retinal neurons and glial culture by immunofluorescence staining: **(a)** Contactin1, red **(b)** Prion, red **(c)** Caspr1, green **(d)** Caspr2, red. **(C)** Representative 60× DIC combined with NeuN (green) immunostained images showing length of neurite outgrowth (red box) in retinal neurons cultured in 5 mM glucose DMEM, 25 mM glucose DMEM, or 25 mM glucose Neurobasal A medium. **(D)** Quantification shows a significant increase in neurite length in 25 mM glucose-containing DMEM and Neurobasal A medium, as compared to 5 mM glucose DMEM (^∗∗^*p* < 0.01). Neurite outgrowth was measured in 50–100 cells per slide, and experiment repeated in triplicate. *N* = 5.

We successfully isolated retinal neurons from adult goat retina, identified by expression of neuronal markers like Synaptophysin, NCAM1 and NeuN (Figure 2). Mammalian neuronal cultures are typically grown in high glucose-containing media based on DMEM or Neurobasal, with addition of supplements to optimize neuronal survival (Tabata et al., 2000; Liu et al., 2013). To study the effect of different glucose concentrations and media compositions on adult neurons, we cultured them in 5 mM and 25 mM glucose-containing DMEM and Neurobasal-A medium (25 mM glucose). High glucose DMEM and Neurobasal-A media differ primarily in osmolarity, and in the addition of serum for DMEM versus serum-free supplements for Neurobasal-A. After 1–2 days, some cells (between 0.9 ± 1.7 and 3.6 ± 2.7 cells per field) were observed adhering to the plate surface, and cell body was predominantly small and round (Figure 1A and Supplementary Table S2). After 3–4 days, cells displayed axon-like projections. The projection-forming process was hastened in cells cultured in hyperglycemic conditions as compared to cells in normoglycemic conditions (Figure 1B). After 5–6 days, projections increased in length, and were on average about 1-2 folds longer than cell body (Figure 1C). At 7–8 days, retinal cells continue to grow in culture (Figure 1D). After 10–11 days, axon-like projections were prominent in length, gradually forming complex networks (Figure 1E).

### Hyperglycemia Does Not Affect Neuronal Viability

A greater number of adherent cells per field were observed in cells cultured in hyperglycemic conditions, as compared to normoglycemic conditions between 4 and 8 days in culture. However, 10 days post primary culture initiation, neuronal cell count appeared to decrease under hyperglycemic conditions (Supplementary Table S2). In order to evaluate possible cytotoxic effects of hyperglycemia, MTT assay was performed. Results of this assay clearly indicate that hyperglycemia does not affect neuronal viability (*P* > 0.05 for 5 mM vs. 25 mM glucose DMEM and Neurobasal-A, Supplementary Figure S3).

### Retinal Neurons Express Cell Adhesion Molecules Expressed in Brain

Immunofluorescence staining was performed to estimate the percentage of cells expressing neuronal and glial markers. Expression of CAMs was examined by staining with primary antibodies against specific CAMs. Retinal cells cultured for 10 days showed 46% of cells expressing neuronal markers like Synaptophysin and NeuN (Figures 2Ab,c). Expression of neuronal CAMs like NCAM1, Contactin1, Prion, Caspr1, and Caspr2 was also observed (Figures 2Aa,Ba–d). Glial marker GFAP was expressed by 15% of cultured cells (Figure 2Ad). The percentage of neurons and glial cells are representative of cells cultured in Neurobasal-A medium containing 25 mM glucose; the numbers did not vary significantly with different media or glycemic conditions.

### Hyperglycemia Increases Neurite Length in Retinal Neurons

Next, we studied the effect of different glucose concentrations and media compositions on neurite outgrowth. Under hyperglycemic conditions, retinal neurons had longer neurite outgrowth (DMEM 18.5 ± 0.63 μm; Neurobasal-A 18 ± 0.45 μm) compared to those cultured in normoglycemic conditions (12.6 ± 0.99 μm) (Figure 2). This suggests that glucose aids neurite extension/growth (*P* < 0.01 for 5 mM vs. 25 mM glucose-containing media, Figure 2D). Our studies reveal that hyperglycemia significantly enhances neurite extension in primary cultures of adult retinal neurons.

### Expression Patterns of Cell Adhesion Molecules in Retina and Brain

**FIGURE 3:**
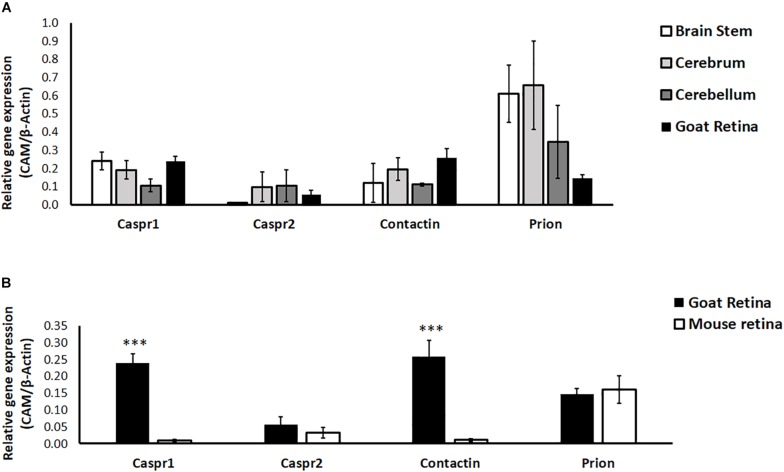
**(A)** Relative expression of specific cell adhesion molecules (Caspr1, Caspr2, Prion, Contactin1) in goat retina and different parts of the brain (cerebrum, cerebellum and brain stem) was quantified by RT-PCR. *N* = 3–4. **(B)** Relative expression of specific cell adhesion molecules (Caspr1, Caspr2, Prion, Contactin1) in goat and BALB/c mouse retina was quantified by RT-PCR. Similar expression levels of Caspr2 and Prion were observed in goat and mouse retina, whereas levels of Caspr1 and Contactin1 were significantly high in goat retina (^∗∗∗^*p* < 0.001 for goat retinal Caspr1 and Contactin1 compared to mouse retina). *N* = 4–5.

Since most studies in neurobiology utilize early postnatal neurons isolated from rodents, we examined the relative expression of CAMs in goat and mouse retina. Further, neuronal CAMs are more widely studied in the brain; hence we compared the relative expression of CAMs in different regions of goat brain (cerebrum, cerebellum and brain stem) with their expression in retina. Results of RT-PCR show similar expression levels of Caspr1, Caspr2, Contactin1, and Prion in retina compared with expression of these proteins in brain regions like cerebrum, cerebellum and brain stem (*P* > 0.05 for all CAMs, Figure 3A). In goat and mouse retinal tissues similar levels of Caspr2 and Prion were expressed. However, expression of Caspr1 and Contactin1 was significantly higher in goat retina relative to mouse retina (*P* < 0.001 for Caspr1 and Contactin1, Figure 3B).

### Hyperglycemia Affects Expression and Transcriptional Regulation of Cell Adhesion Molecules

**FIGURE 4:**
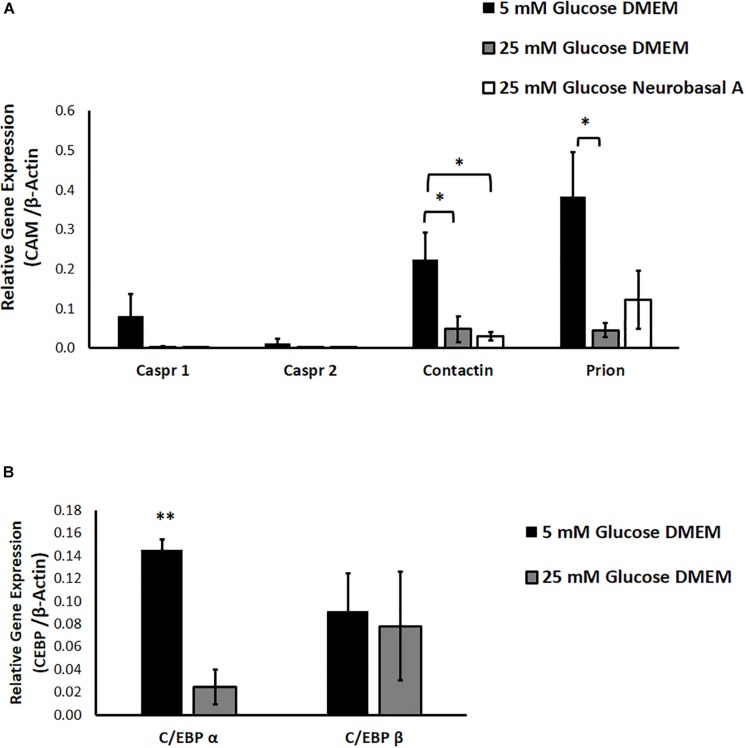
**(A)** The effect of hyperglycemia and media composition on expression of specific cell adhesion molecules in cultured retinal cells was quantified by RT-PCR. In 25 mM glucose DMEM and Neurobasal A medium, Contactin1 expression was significantly downregulated. In 25 mM DMEM, Prion expression was significantly downregulated. ^∗^*p* < 0.05 compared to 5 mM glucose medium. *N* = 4–5. **(B)** The effect of hyperglycemia on expression of transcription factors C/EBP α and C/EBP β in cultured retinal cells was quantified by RT-PCR. In 25 mM glucose medium, expression of C/EBP α was significantly downregulated. ^∗∗^*p* < 0.01 compared to 5 mM glucose medium. *N* = 3.

We studied the effect of different glucose concentrations and media compositions on expression of CAMs. Results of RT-PCR show that expression of Prion and Contactin1 is significantly downregulated under hyperglycemia (*P* < 0.05 for Contactin1 and Prion). Caspr1 expression shows a clear trend toward downregulation in hyperglycemia, while Caspr2 expression was low in normoglycemia and did not change significantly under hyperglycemic conditions. Media composition did not have any significant effect on expression of neuronal CAMs (Figure 4A).

We also explored the contribution of glucose homeostasis-sensitive transcription factors, CCAAT/enhancer binding protein isoforms C/EBP-α and C/EBP-β toward regulation of neuronal CAM expression. Using Alibaba2 program, we predicted that C/EBP-α and C/EBP-β have binding sites in goat neuronal CAM genes (Caspr1, Caspr2, Contactin1 and Prion) within −600 bp proximal to the promoter region (Supplementary Table S3). “Start” and “Stop” columns indicate specific locations of putative binding sites for C/EBP-α and C/EBP-β upstream of ATG-translation initiation codon. We studied the effect of hyperglycemia on expression of C/EBP-α and C/EBP-β. RT-PCR indicated that hyperglycemia significantly downregulates expression of C/EBP-α, while expression of C/EBP-β remains unchanged (*P* = 0.002 for C/EBP-α, Figure 4B).

## Discussion

Much is known about individual disease states such as diabetes, metabolic syndrome, and dementia; nevertheless, there is a need to identify where and how their pathophysiology intersects. Our study evaluates effects of hyperglycemia on expression and transcriptional regulation of CAMs in retinal neurons cultured from adult goat. We show for the first time that hyperglycemia significantly enhances neurite outgrowth, with downregulation of CAMs in cultured retinal neurons (Figures 2C,D, 4A). Neuronal CAMs have been well-characterized in the brain for their role in regulating neurite outgrowth (Santuccione et al., 2005; Devanathan et al., 2010) and in the nodal/paranodal domain organization of myelinated axons (Peles and Salzer, 2000). However, the function of CAMs has not been elucidated in the retina, which consists of non-myelinated neurons. Here, we demonstrate that specific CAMs (Caspr1, Caspr2, Contactin1 and Prion) are expressed in the retinal tissue at levels comparable to their expression in brain (Figure 3A), and that their expression is downregulated under hyperglycemic conditions (Figure 4A).

Caspr1 and Contactin1 typically form a complex at the paranodal junction of myelinated neurons in the CNS. The absence of Caspr1 leads to mislocalization of Contactin1 and its exclusion from the paranodes (Bhat et al., 2001), while the absence of Contactin1 prevents delivery of Caspr1 to the axonal membrane (Boyle et al., 2001). We have previously demonstrated that Prion protein directly binds to Caspr1, protecting it from proteolysis. Deficiency of Prion results in reduced levels of Caspr at the neuronal membrane and enhanced neurite extension *in vitro* (Devanathan et al., 2010). In our current study we go one step further, and show that hyperglycemia leads to a significant reduction in levels of specific neuronal CAMs, while enhancing neurite length (Figures 2C,D, 4A). Neurite extension in adult neurons is also believed to be enhanced by an IL-1β-dependent pathway (Saleh et al., 2013). Since hyperglycemia is known to increase levels of pro-inflammatory cytokines such as IL-1β and TNF-α in the diabetic retina (Busik et al., 2008; Chakravarthy et al., 2016), these factors presumably exert their effect on neuritogenesis as observed in our study (Figures 2C,D).

Neuronal CAM expression can be modulated in different ways including transcriptional regulation. The C/EBP constitute a family of transcription factors sensitive to changes in glucose homeostasis (Matsusue et al., 2004; Schroeder-Gloeckler et al., 2007). Among six C/EBP isoforms, C/EBP-α, β and δ are enriched in CNS neurons, and have been implicated in neuronal development, survival and neurogenesis (Ménard et al., 2002; Ramji and Foka, 2002; Paquin et al., 2005). C/EBP-α expression is downregulated, while C/EBP-β is upregulated in animal models of diabetes (Arizmendi et al., 1999). In our study, we observe a similar downregulation of C/EBP-α expression in cultured retinal cells under hyperglycemic conditions (Figure 4B). We also identified binding sites for C/EBP-α and β upstream of the promoter in goat neuronal CAM genes, Contactin1, Caspr1, Caspr2 and Prion (Supplementary Table S3). C/EBPs act as transcriptional enhancers or repressors depending on the C/EBP isoform, target gene and cell-type. We propose that hyperglycemia represses expression of C/EBP α, thereby diminishing its transcription-activating effect on CAMs, which in turn leads to downregulation of CAM expression in retinal neurons (Supplementary Figure S4).

A unique advantage of studying neurodegenerative changes in adult retina is easier accessibility to CNS neurons, and rapid detection of neuronal abnormalities in animal models using non-invasive techniques such as multifocal electroretinography, ultra-widefield fundus imaging, and spectral-domain OCT (Cheung et al., 2017; Chakravarthy and Devanathan, 2018). Moreover, administration of experimental drugs to the retina in animal models is relatively easier, making it a robust tool for preclinical studies (Edelhauser et al., 2010). Retinal and brain neurodegenerative diseases although affecting different parts of the CNS, appear to involve similar pathogenic mechanisms like oxidative stress, low-grade chronic neuroinflammation, disruption of blood-brain or blood-retinal barrier, and vascular abnormalities (London et al., 2013; Colligris et al., 2018). Increasing clinical and preclinical evidence points to associations between metabolic dysregulation and neurodegenerative brain disease (Weinstein et al., 2015; Arnold et al., 2018; Ogama et al., 2018).

Culture of primary neurons is an indispensable method to study pathological responses of CNS neurons to metabolic conditions or inflammatory environments associated with neurodegenerative diseases. However, most *in vitro* experiments in neurobiology employ neurons isolated from rodents because of advantages such as ease of availability, well-characterized genetics, and availability of tissue from transgenic animals. Moreover, primary retinal cultures are typically generated from early postnatal rat or mouse pups. There are several disadvantages to using rodent tissue for cell culture. Cell yields from multiple animals typically permit a single experiment, or may be used in studies involving single cell electrophysiology. Compared to studies in rodents which yield lower retinal cell numbers, we successfully obtained 20 million cells from retina isolated from one goat eye which is suitable for several experiments over 3–10 days, and greatly reduces the number of animals needed. Once cultured, adult retinal neurons can be subjected to genetic manipulation, or physiological, biochemical and pharmacological procedures for functional characterization experiments. An additional advantage is that these animals were not sacrificed exclusively for the purpose of isolating CNS neurons, since animal parts were subsequently sold at the abattoir for other purposes.

Another important concern is that although mouse and human genomes are 85% similar, mouse models often do not accurately predict human pathophysiology leading to failure of clinical trials based on preclinical validation in mice, especially in the field of neurodegenerative diseases (Gordon et al., 2007; Duyckaerts et al., 2008; Dawson et al., 2010). Goats and cattle are considered phylogenetically distant from humans and rodents (Kim et al., 2017); however, elevated rate of evolution in rodents relative to other mammals is believed to result in higher amino acid sequence identity between human and ruminant proteins as compared to human and rodent proteins (Bovine Genome Sequencing and Analysis Consortium et al., 2009). Indeed, a basic sequence comparison among orthologous CAMs across species using Clustal Omega demonstrates higher sequence similarity between goat and human relative to rodent proteins, as observed in previous studies (Bovine Genome Sequencing and Analysis Consortium et al., 2009). Thus, *in vitro* studies of protein interactions and signaling in cells cultured from goat or cow could facilitate identification of novel functionally important pathways in higher mammals.

## Conclusion

To the best of our knowledge, no other study has been conducted using a similar approach to culture retinal neurons from adult goat. Higher mammals such as pigs have similar vasculature and retinal structures as humans, while dogs develop morphological lesions most similar to diabetic human retina (Lai and Lo, 2013). While the neural retina in all mammals contains distinct classes of interneurons, species-specific functional differences have been found in overtly similar cell types (Dacey et al., 2003; Dhande et al., 2019). Further, traditional laboratory-bred animals have a limited number of alleles which does not reflect the genetic diversity and natural variation found in human populations. Therefore, *in vitro* models using higher mammals can provide an important platform for investigating neuronal responses that may not be revealed by rodent-derived cells. Additionally, exploring metabolic and signaling mechanisms in adult retinal neurons may provide vital insights that can be translated to neurodegenerative processes in the entire CNS. The unique model of adult goat retinal culture described in our study can be used to perform detailed investigations into effects of hyperglycemia on interactions between specific CAMs expressed on distinct retinal neuronal types, as well as related changes in downstream signaling to elucidate functions of CAMs in adult retina.

## Data Availability

All datasets generated for this study are included in the manuscript and/or the Supplementary Files.

## Ethics Statement

The animal study was reviewed and approved by Institutional Animal Ethics Committee (IAEC), Sri Ramachandra Medical College and Research Institute. Written informed consent was obtained from the owners for the participation of their animals in this study.

## Author Contributions

SS and HC performed the research, analyzed the data, and wrote the manuscript. GS performed the research and analyzed the data. VD designed the research, contributed reagents and analytic tools, wrote the manuscript, and had full access to all data in the study. All authors took responsibility for the integrity of the data and accuracy of data analysis.

## Conflict of Interest Statement

The authors declare that the research was conducted in the absence of any commercial or financial relationships that could be construed as a potential conflict of interest.
