# Supplementary material for: Adult Goat Retinal Neuronal Culture: Applications in Modeling Hyperglycemia
Source: Front Neurosci. 2019 Sep 16;13:983. doi: 10.3389/fnins.2019.00983 (PMC6756134; doi:10.3389/fnins.2019.00983)
Supplement: Supplementary file 1 [file Data_Sheet_1.pdf]

Table S1. Quantitative RT-PCR primers list

Animal	Gene name	Forward	Reverse
Goat	Caspr1	AAGACCCACTTCCGAACGCC	TGGCATGGCTCCGCAGTTAG
	Caspr2	CGGCCTCTTTCCCAAACCCA	GGCCCGTTCCCGACATCAAA
	Prion	GAAGCGACCAAAACCTGGCG	TGAGGTGGATAGCGGTTGCC
	Contactin	CTTGGAGCCGTGGTTCAGACA	AATCCACTGCTCTTGCTGCCT
	C/EBP α	CTGCCCGAACAGAACAGCGT	ATCGGAGCGGTGAGTTTGCG
	C/EBP β	GCCCGCCCGTGGTGTTATTTA	TGTACACACGCGTTCAGCCA
	GAPDH	CCGTAACTTCTGTGCTGTGCC	CGTTCACTCCGACCTTCACCA
	β-Actin	CTGGACTTCGAGCAGGAGATG	GTTTCGTGAATGCCGCAGGA
Mouse	Caspr1	CGTTGGTCAGCTCCGCATGA	GCGACCATCCACTGATGCCA
	Caspr2	CAAAGCAGGAGGAGCTTGGCT	CGGGCCAGTGGTACTCCAAA
	Prion	GGCCTTGGTGGCTACATGCT	TAGCGGTCCTCCCAGTCGTT
	Contactin	AGATGGTCCCCAATTCAGCGG	TGTCACACGCAGTCGGGTCTA
	β-Actin	ACTCTGTGTGGATCGGTGGC	CAGCTCAGTAACAGTCCGCCT
