# Supplementary material for: Adult Goat Retinal Neuronal Culture: Applications in Modeling Hyperglycemia
Source: Front Neurosci. 2019 Sep 16;13:983. doi: 10.3389/fnins.2019.00983 (PMC6756134; doi:10.3389/fnins.2019.00983)
Supplement: Supplementary file 2 [file Data_Sheet_2.pdf]

Culture Media	Day 2	Day 4	Day 6	Day 8	Day 10
5 mM Glucose DMEM	0.9 ± 1.7	1.9 ±1.7	13.3 ± 6.6	19.9 ± 16.1	72.2 ± 45.9
25 mM Glucose DMEM	1.4 ± 1.8	9.5 ± 9.8	16.9 ± 17.0	141 ± 89	134 ± 117
25 mM Glucose Neurobasal A	3.6 ± 2.7	10.2 ±2.8	103 ± 125	147 ± 118	68.7 ± 74.2

Table S2. Cell counts per field (results expressed as mean ± SD) for each group. 6-10 random fields were examined under 10X magnification. Retinal neurons were cultured in 5 mM glucose DMEM, 25 mM glucose DMEM, or 25 mM glucose Neurobasal A medium for up to 10 days.
