# Supplementary material for: Adult Goat Retinal Neuronal Culture: Applications in Modeling Hyperglycemia
Source: Front Neurosci. 2019 Sep 16;13:983. doi: 10.3389/fnins.2019.00983 (PMC6756134; doi:10.3389/fnins.2019.00983)
Supplement: Supplementary file 3 [file Data_Sheet_3.pdf]

Gene	Sequence recognized	Transcription Factor	Start	Stop
Contactin1	**ACTAATAATT**	C/EBP α	-550	-541
Contactin1	**TTATGCAGGT**	C/EBP β	-85	-76
Caspr1	**AGGCGGGGGA**	C/EBP α	-387	-378
Prion	**AGCTATTCAG**	C/EBP α	-559	-550
Caspr2	**CACGCGGGCG**	C/EBP α	-48	-39

Table S3. Binding sites for the transcription factors C/EBP α and C/EBP β upstream of goat CAM promoters, predicted using Alibaba2 software, Version 2.1, Germany.
