# Supplementary figures and images for: Adult Goat Retinal Neuronal Culture: Applications in Modeling Hyperglycemia
Source: Front Neurosci. 2019 Sep 16;13:983. doi: 10.3389/fnins.2019.00983 (PMC6756134; doi:10.3389/fnins.2019.00983)

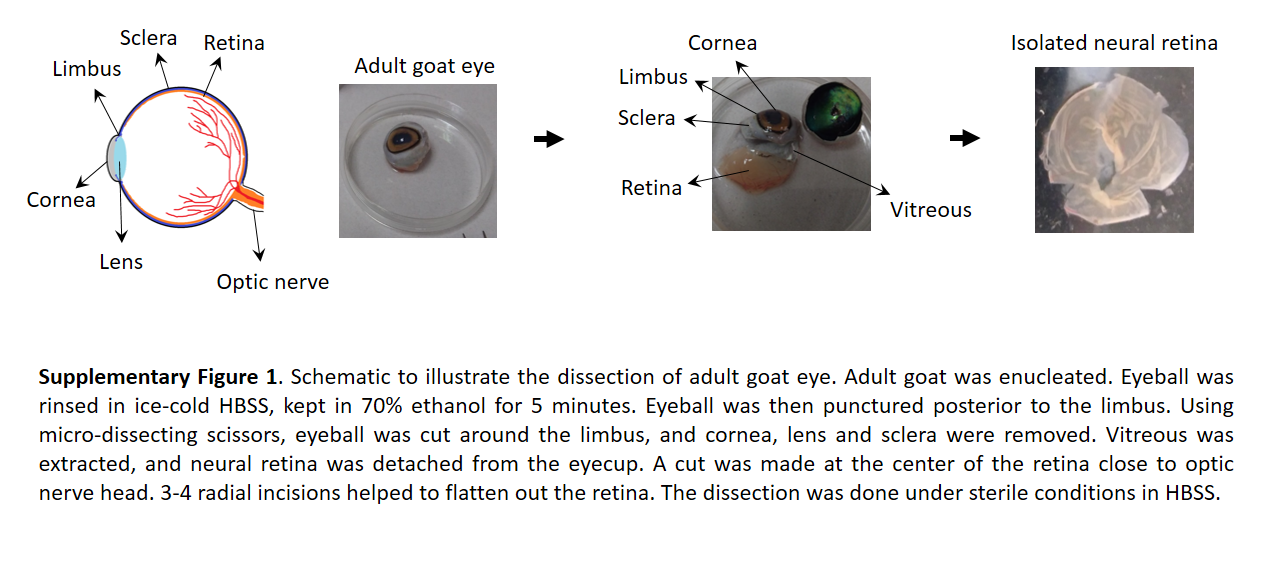

Supplement: Supplementary file 4 [file Image_1.tif]

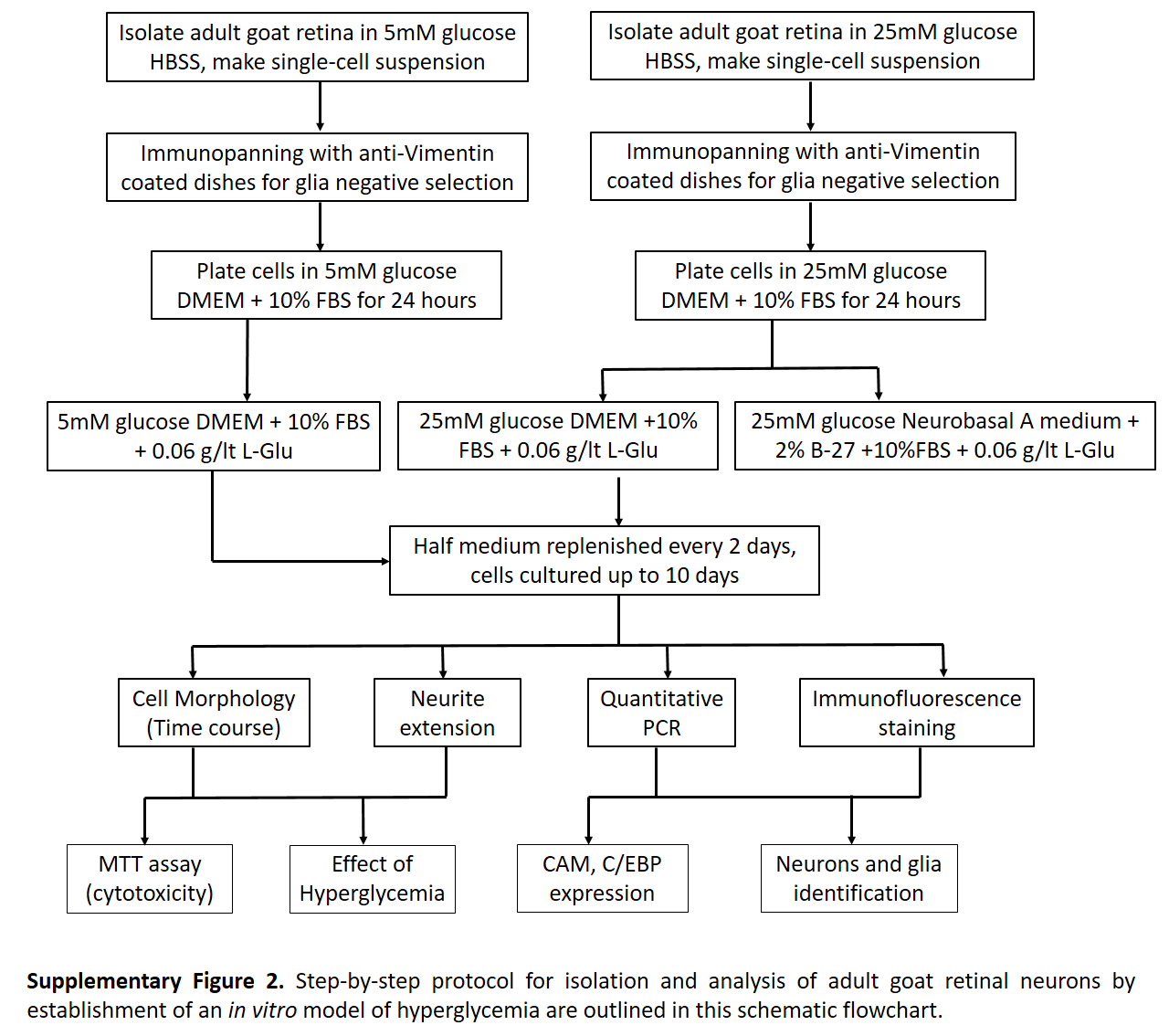

Supplement: Supplementary file 5 [file Image_2.TIF]

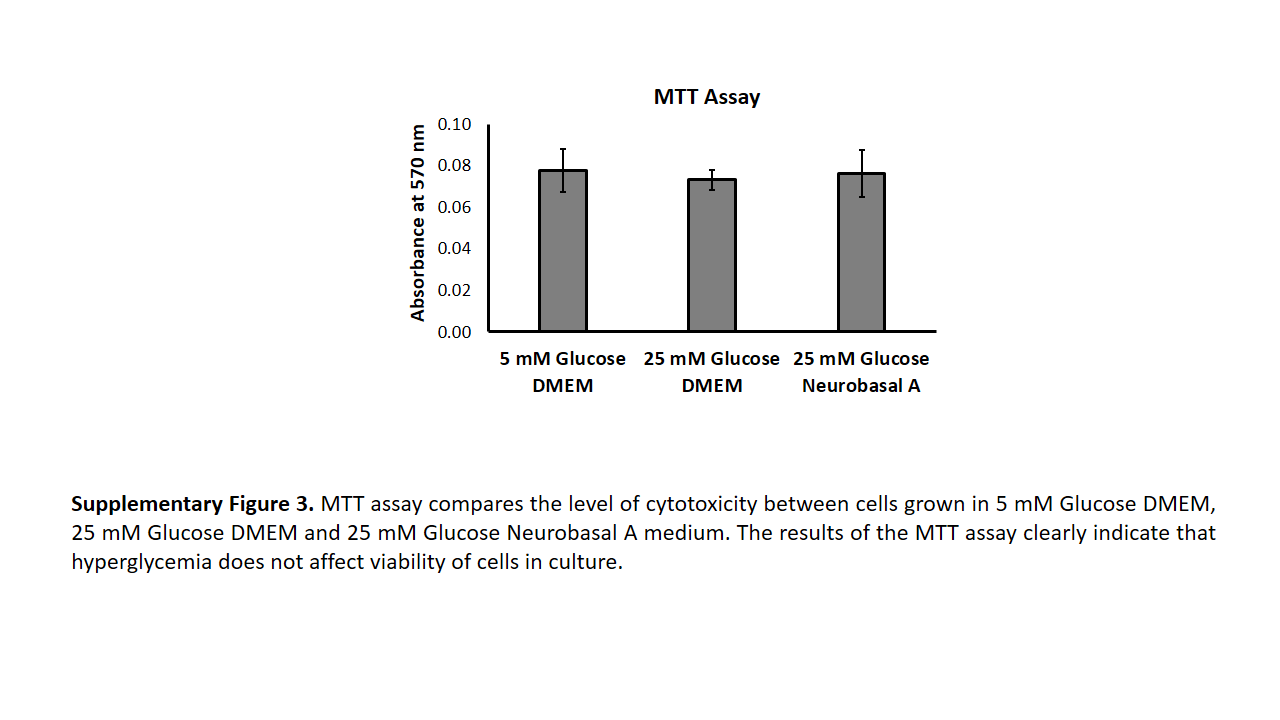

Supplement: Supplementary file 6 [file Image_3.tif]

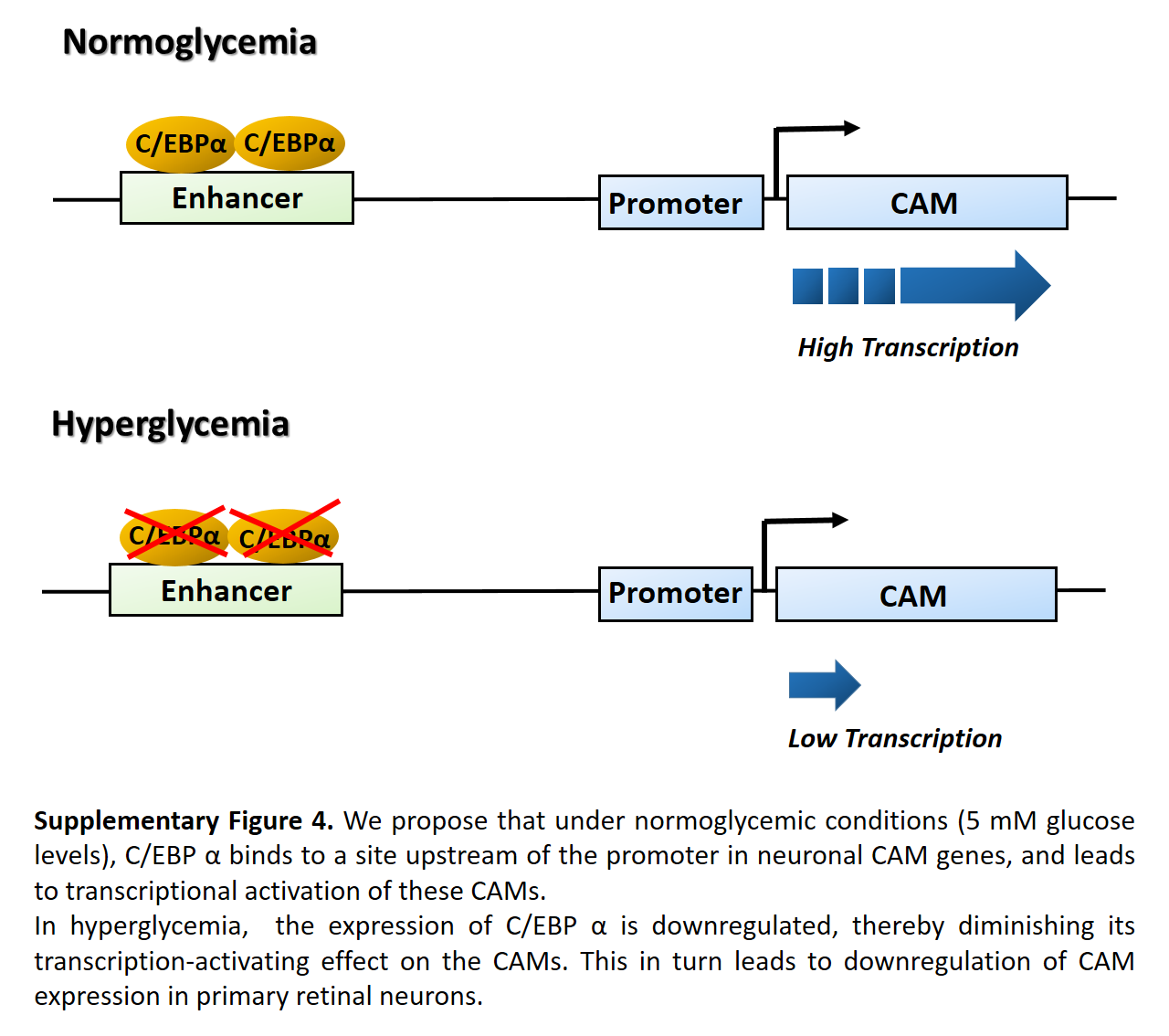

Supplement: Supplementary file 7 [file Image_4.tif]
